# *In vitro* antibacterial activity of *Tabernaemontana alternifolia* (Roxb) stem bark aqueous extracts against clinical isolates of methicillin resistant *Staphylococcus aureus*

**DOI:** 10.1186/1476-0711-12-26

**Published:** 2013-09-25

**Authors:** Nachiket P Marathe, Mandar H Rasane, Himanshu Kumar, Ankur A Patwardhan, Yogesh S Shouche, Sham S Diwanay

**Affiliations:** 1 Microbial Culture Collection, National Centre for Cell Science, Ganeshkhind, Pune 411007, India; 2 Department of Biodiversity, Abasaheb Garware College, Karve Road, Pune 411004, India; 3 Department of Microbiology, Abasaheb Garware College, Karve Road, Pune 411004, India

**Keywords:** *Tabernaemontana alternifolia* (Roxb), Anti-MRSA, Cytotoxicity, Plant extract, Antimicrobial

## Abstract

**Background:**

The rise of antibiotic resistance among methicillin resistant *Staphylococcus aureus* (MRSA), have caused concerns for the treatment of MRSA infections. Hence, search for an alternative therapy for these infections is inevitable. Folk Indian medicine refers to the use of leaf and stem bark powder of *Tabernaemontana alternifolia* (Roxb) in treatment of skin infections, but no scientific report establishes its antibacterial activity.

**Methods:**

Direct aqueous extracts and sequential aqueous extracts of the stem bark of *T. alternifolia* (using petroleum ether and ethyl acetate as other solvents) were prepared by soxhlet extraction. The antibiotic sensitivity profiles of the clinical isolates were determined against 18 antibiotics using disc diffusion method. The isolates were identified by 16S rRNA gene sequencing. The methicillin resistance among *S. aureus* (MRSA) was confirmed by PCR amplification of *mecA* gene. The disc diffusion method was used to determine the antibacterial activity of the extracts. The micro-dilution method was used to determine the minimum inhibitory concentration (MIC) of the extract against the test organism. To further evaluate the therapeutic potential of the extract, cell cytotoxicity was checked on Vero cells by MTT assay. Chemical profiling of the extract was done by HPTLC method.

**Results:**

The aqueous extracts of *T. alternifolia* stem bark exhibited antibacterial activity against Gram-positive microorganisms, particularly against clinical isolates of MRSA and vancomycin resistant *S. aureus* (VRSA). The minimum inhibitory concentration (MIC) of extract against the isolates ranged from 600–800 μg/ml. The extract did not exhibit cytotoxic activity against Vero cells even at the concentration of 4 mg/ml. The chemical profiling revealed presence of alkaloids, flavonoids, coumarins, saponins and steroids. Petroleum ether and ethyl acetate extracts did not exhibit antibacterial activity.

**Conclusion:**

Our results offer a scientific basis for the traditional use of *T. alternifolia* in the treatment of skin infections, showing that the plant extract has an enormous potential as a prospective alternative therapy against MRSA skin infections. The present study lays the basis for future studies, to validate the possible use of *T. alternifolia* as a candidate in the treatment of MRSA infections.

## Introduction

Infectious diseases are one of the world’s leading causes of premature deaths [1]. Antibiotics which are widely used for the treatment of infectious diseases are under constant threat due to the emergence of antibiotic resistant pathogens such as methicillin resistant *Staphylococcus aureus* (MRSA), multidrug resistant *Pseudomonas aeruginosa*, vancomycin resistant *Enterococcus* (VRE) and multidrug resistant *Mycobacterium tuberculosis*[2-6]. Among these, MRSA has emerged as agents causing increasing threat of nosocomial infections, more people have died of MRSA infection in US hospitals than of HIV/AIDS and tuberculosis combined during 2007–2008 [7,8]. Hence, search for novel antimicrobial compounds or alternative therapy for these infections is inevitable.

Plant based medicines are widely used and form an integral part of primary health care in many developing countries across the globe [9-12]. Recently plants have been explored to obtain crude natural extracts for testing and further refinement to develop effective antimicrobial drugs. In India there are different systems of medicine practiced like Ayurveda, Siddha, Unani, Amchi and local health traditions, which utilize a large number of plants or herbs for the treatment of human diseases [13].

Many plant species widely explored for antimicrobial compounds fall into the family Apocynaceae. Within this family, the genus *Tabernaemontana* is well documented for its biological activities such as antioxidant [14], anticancer [15-17], antifertility/contraceptive [18,19], anti-pyretic and anti-inflammatory [17,20], anti-mycobacterial [14], and antimicrobial agent [21-23].

*T. alternifolia* (Roxb), also known as *Ervatamia heyneana* (Wall) or *T. heyneana* (Wall) is endemic to India and found in Goa, Karnataka, Kerala, Maharashtra and Tamil Nadu states [24]. Traditionally a therapeutic preparation made from leaf and stem powder of *T. alternifolia* (Roxb) in combination with stem bark of *Ficus racemosa, Ficus benghalensis, Madhuca longifolia and Strychnos nux-vomica* is used to treat skin infections [25]. The other plants, except *T. alternifolia*, used in this preparation have reported antibacterial activity, but despite the traditional use of *T. alternifolia* in treatment of skin infections, no scientific report has focused on establishment of the antimicrobial activity of the plant against pathogen [26-29]. We therefore, under took this study to evaluate the antimicrobial potential of the extracts of stem bark of *T. alternifolia* against pathogens causing skin infections, especially MRSA and VRSA.

## Methods

### Collection of plant material

Stem bark was collected in sterile plastic bags from Tamhini Ghat, a part of Western Ghats (18°28′21″ N, 73°25′07″ E) near Pune, Maharashtra, India. The plant identity was validated at the Department of Biodiversity, Abasaheb Garware College, Pune.

### Preparation of extracts

The aqueous extracts of stem bark were prepared as follows: Direct aqueous extract (DAE): The stem bark was dried at 55°C overnight. Twenty gram of dried stem bark was subjected to hot extraction using soxhlet apparatus with 200 ml of distilled water as solvent for 6 hours at 100°C (boiling water).

Sequential aqueous extract (SAE): Twenty gram of dried stem bark was subjected to hot extraction using soxhlet apparatus first with 200 ml petroleum ether as a solvent for 2 hours followed with ethyl acetate for 2 hours and finally with distilled water for 6 hours at 100°C.

The crude extracts were concentrated at 55°C in a clean and sterile glass petri plate. Final solution of 100 mg/ml was prepared in sterile distilled water for aqueous extracts and in dimethyl sulfoxide (DMSO) for ethyl acetate and petroleum ether extracts. The extracts were filter sterilized using Millipore 0.22 μm filter and stored at 4°C.

### Test organisms and antibiotic sensitivity determination

Clinical isolates from skin and soft tissue infections were obtained from Deenanath Mangeshkar Hospital, Pune. These clinical isolates were collected by the hospital as a part of the standard practice and made available for the study. The type strains of *Bacillus subtilis* (ATCC 6633), *Staphylococcus aureus* (ATCC 6538P), *S. epidermidis* (ATCC 12228), *Escherichia coli* (ATCC 8739), methicillin resistant *S. aureus* (ATCC 43300) were obtained from NCIM, National Chemical Laboratory, Pune.

Antibiotic sensitivity pattern of clinical isolates was determined by standard disc diffusion method according to the standards prescribed by Clinical and Laboratory Standards Institute (CLSI) (former NCCLS) [30].

### DNA extraction and PCR

The genomic DNA extraction was carried out from freshly grown bacterial cultures using Qiagen blood and tissue DNA extraction kit (Qiagen, Madison USA), following the manufacturer’s instructions. The isolates were identified by 16S rRNA gene sequencing method as described earlier [31]. PCR amplification of 16S rRNA gene was done using primers 27F 5′-AGAGTTTGATCATGGCTCAG-3′ and 1488R 5′-CGGTTACCTTGTTACGACTTCACC-3′. PCR amplification involved a GeneAmp PCR system 9700 (Applied Biosystems Inc. USA). The PCR for *mecA* gene was carried out as described earlier [32]. The DNA sequencing was done using BigDye 3.1 sequencing terminator kit and ABI 3730xl DNA analyzer (Applied Biosystems Inc. USA).

### Antibacterial activity using disc diffusion method

Antibacterial activity of the extract was determined as per CLSI guidelines (formerly NCCLS guidelines) [30]. Briefly, Whatmann filter paper no. 1 discs were prepared (diameter 6 mm). Discs were impregnated with 20 μl of 100 mg/ml extract per disc and dried at room temperature. Bacterial suspensions were prepared by suspending overnight grown culture in sterile normal saline. The turbidity of bacterial suspensions was adjusted to 2x10^6^ cfu/ml and 100 μl of suspension was spread on Muller-Hinton agar plate (HiMedia Laboratories, India). The discs impregnated with extract were placed on plate. The plates were incubated at 37°C for 24 hours and the zones of inhibition were measured. Ciprofloxacin discs (5 μg/disc) (HiMedia Laboratories, India) were used as a positive control for growth inhibition.

### Determination of minimum inhibitory concentration

Minimum inhibitory concentration (MIC) was determined by the microdilution broth method [33]. The plant extracts were serially diluted with Mueller–Hinton broth (HiMedia laboratories, India) to obtain the desired concentrations of 0.1 to 2 mg/ml. Bacterial suspensions were prepared in the similar manner as described in disc diffusion assay. One hundred microliter of the suspension was added to serially diluted extract. The inoculated test tubes were incubated at 37°C under aerobic conditions. Ciprofloxacin (HiMedia Laboratories, India) was used as a positive control for growth inhibition, in concentration ranging from 0.05 μg/ml to 10 μg/ml. Turbidity was checked after 24 hours of incubation. The lowest concentration of the extract that produced no visible growth when compared to the control (tube containing no inoculum) was considered as MIC.

### Stability of antibacterial activity

At a regular interval of three days, the antibacterial activity of the extract was checked against MRSA (DMH4) isolate by well diffusion method for a period of 2 months [34]. Briefly, wells of diameter 8 mm were bored in the pre seeded Mueller–Hinton agar plates using a cork borer and 100 μl of SAE (20 mg/ml) was added in the well. The plates were incubated at 37°C and the zones of inhibition were measured after 24 hours. The zone of inhibition after every 3 days was compared with the zone of inhibition on day 1 (first reading) to check the stability of the antibacterial activity of the extract.

### Cell cytotoxicity of the aqueous extracts

Cytotoxicity of extract on Vero cells was measured by microculture tetrazolium (MTT) assay [35]. For the assays, 96-well microplate was seeded with 100 μl medium containing 10,000 Vero cells in suspension. After 24 hr incubation and attachment, the cells were treated with different dilutions of DAE as well as SAE. Various dilutions of the extracts were prepared from the stock solution (100 mg/ml), and incorporated in cell culture (final concentrations ranging from 1 to 4 mg/ml), in quadruplicate. Cell viability was determined after 24 hour incubation, by adding tetrazolium salt (Sigma Aldrich, USA) and reading the absorbance at 570 nm with a ELISA plate reader Spectra MAX250 (Molecular Devices, USA). Tetrazolium salts are cleaved to formazan dye by cellular enzymes (only in the viable cells). The level of absorbance directly correlates to the metabolically active cells.

### Chemo-profiling of the extract

Chemical analysis of SAE was done by HPTLC (Camag, Switzerland) Sequentially: Applicator – Linomat IV, Scanner III; Plate was developed in a twin tray chamber. Solvent system and spraying reagents were used as described earlier [36].

## Results

### Identification and antibiotic sensitivity pattern of pathogenic isolates

A total of 14 isolates were obtained from patients with skin infections. Based on 16S rRNA gene sequence, the isolates were identified as *Pseudomonas aeruginosa* (DMH 1), *Staphylococcus aureus* (DMH 2 to DMH 8 and DMH 10 to DMH 14), and *Escherichia coli* (DMH 9). The 16S rRNA gene sequences of isolates are deposited at GenBank under accession numbers HM559231 to HM559244. The drug sensitivity pattern, represented in Table 1 revealed that all the isolates were resistant to at least 4 different antibiotics, showing that all isolates are multi-drug resistant. Eleven out of twelve *S. aureus* strains were methicillin resistant (MRSA). The presence of *mecA* gene in the isolates, as demonstrated by PCR and sequencing, confirmed the methicillin resistant nature of the isolates which was earlier observed by disc diffusion assay. The *mecA* gene is the structural gene present in MRSA for penicillin binding protein 2a, which confers resistance to most of the βeta-lactam antibiotics [32]. Four of the MRSA isolates were also resistant to vancomycin, Table 1, thus making them VRSA.

**Table 1:** Antibiotic sensitivity patterns of the test organisms

**Antibiotics**	**Isolates**													
	**DMH1**	**DMH2**	**DMH3**	**DMH4**	**DMH5**	**DMH6**	**DMH7**	**DMH8**	**DMH9**	**DMH10**	**DMH11**	**DMH12**	**DMH13**	**DMH14**
Ampicillin	R	S	R	R	R	R	R	R	R	R	R	R	R	R
Cefuroxime	R	R	R	R	R	S	R	R	R	S	R	R	R	R
Cephadroxil	R	R	R	R	R	R	R	R	R	S	S	S	S	S
Augmentin	R	S	R	R	R	R	R	R	R	R	R	R	R	R
Penicillin	R	S	R	R	R	R	R	R	R	R	R	R	R	R
Azithromycin	S	R	S	R	R	S	S	S	S	S	S	R	R	S
Erythromycin	R	R	S	R	R	S	S	S	R	S	S	R	IR	S
Cefoperazone	S	S	IR	R	S	S	IR	S	IR	IR	IR	R	S	S
Clarithromycin	R	R	S	R	R	S	S	S	R	S	S	R	IR	S
Ciprofloxacin	S	S	R	R	IR	S	S	S	R	S	IR	R	R	R
Gatifloxacin	S	S	S	S	S	S	S	S	R	S	S	S	S	S
Aztreonam	R	R	R	R	R	R	R	R	R	R	R	R	R	R
Vancomycin	R	S	R	S	S	S	S	S	R	R	S	R	S	R
Doxycycline Hydrochloride	R	S	S	S	S	S	S	S	R	S	S	S	S	S
Norfloxacin	S	S	IR	IR	IR	S	S	S	R	S	IR	IR	R	R
Ofloxacin	S	S	S	S	S	S	S	S	R	S	S	S	S	S
Sparfloxacin	S	R	S	S	S	S	S	S	R	S	S	S	S	S
Methicillin	R	S	R	R	R	R	R	R	R	R	R	R	R	R

Legend: *S* sensitive, *IR* intermediate resistant, *R* resistant.

DMH 1: *Pseudomonas aeruginosa*, DMH 2 to DMH 8: *Staphylococcus aureus*, DMH 9: *Escherichia coli*, DMH 10 to DMH 14: *Staphylococcus aureus.*

### Antibacterial activity and MIC of the aqueous extract against bacterial pathogen

Both the hot aqueous extracts (DAE and SAE) exhibited antibacterial activity against Gram positive organisms such as *B. subtilis*, *S. epidermidis, S. aureus* and MRSA but did not exhibit any antibacterial activity against Gram negative organisms like *E. coli* and *P. aeruginosa*, represented in Table 2. This observation is in accordance with the antimicrobial activity obtained for other plant species from the same genus, for eg., methanolic extracts of *T. chippi* stem bark has reported antimicrobial activity against Gram positive organisms and very weak activity against gram negative organisms [37]. Similarly *T. angulate* and *T. stapfiana* (Britten) have antimicrobial activity against *S. aureus*[22,23].

**Table 2:** **Zones of inhibition and MIC values for *T. alternifolia *stem bark aqueous extracts against the test organisms**

**Isolate**	**Zone of inhibition (mm)**			**Minimum inhibitory concentration (μg/ml)**		
	**DAE**	**SAE**	**Ciprofloxacin (5 μg/disc)**	**DAE**	**SAE**	**Ciprofloxacin**
DMH 2	16	17	27	600	600	0.25
DMH 3	16	16	11	700	650	2
DMH 4	14	14	13	800	800	2
DMH 5	18	19	15	600	600	1.5
DMH 6	13	14	22	800	800	0.25
DMH 7	15	15	21	700	650	0.25
DMH 8	14	14	24	800	800	0.125
DMH 10	15	15	20	700	650	0.25
DMH 11	16	16	16	700	700	1.4
DMH 12	17	17	13	600	600	2.5
DMH 13	16	16	12	700	700	3
DMH 14	14	14	11	800	800	2
ATCC 6633	16	17	25	600	600	0.05
ATCC6538P	17	16	22	600	600	0.15
ATCC12228	17	16	24	600	600	0.1
ATCC 43300	17	17	22	600	600	0.15

Legend: All DMH isolates are *Staphylococcus aureus* among which DMH 3,10,12,14 are VRSA and all others except DMH2 are MRSA. ATCC 6633: *Bacillus subtilis,* ATCC 6538P: *Staphylococcus aureus,* ATCC 12228: *Staphylococcus epidermidis,* ATCC 43300: MRSA.

**Figure 1:**
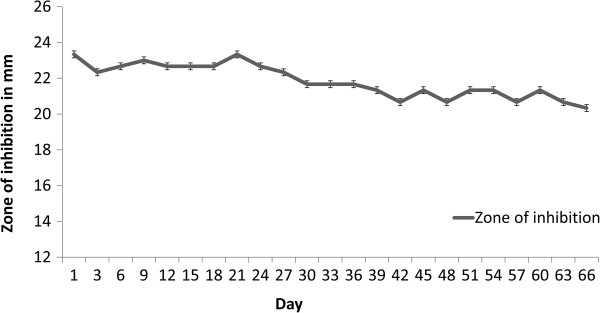
**Consistent antimicrobial activity of SAE (sequential aqueous extract) against DMH 4 (MRSA).** The X axis represents the number of days after preparation of extract and Y axis represents the zone of inhibition (in mm) by well diffusion method.

The values for minimum inhibitory concentrations (MIC) for DAE and SAE against the test organisms are represented in Table 2. The MIC of extracts against MRSA isolates fall in the range of 600–800 μg/ml as determined by broth dilution method. The antibacterial activity of the extract was stable even after 2 months as suggested by consistent zone of inhibition by well diffusion method (Figure 1). Petroleum ether and ethyl acetate extracts did not exhibit any antibacterial activity. The MIC for ciprofloxacin against MRSA isolates ranged from 0.125 μg/ml to 3 μg/ml (Table 2).

### Cell cytotoxicity of the aqueous extracts

**Figure 2:**
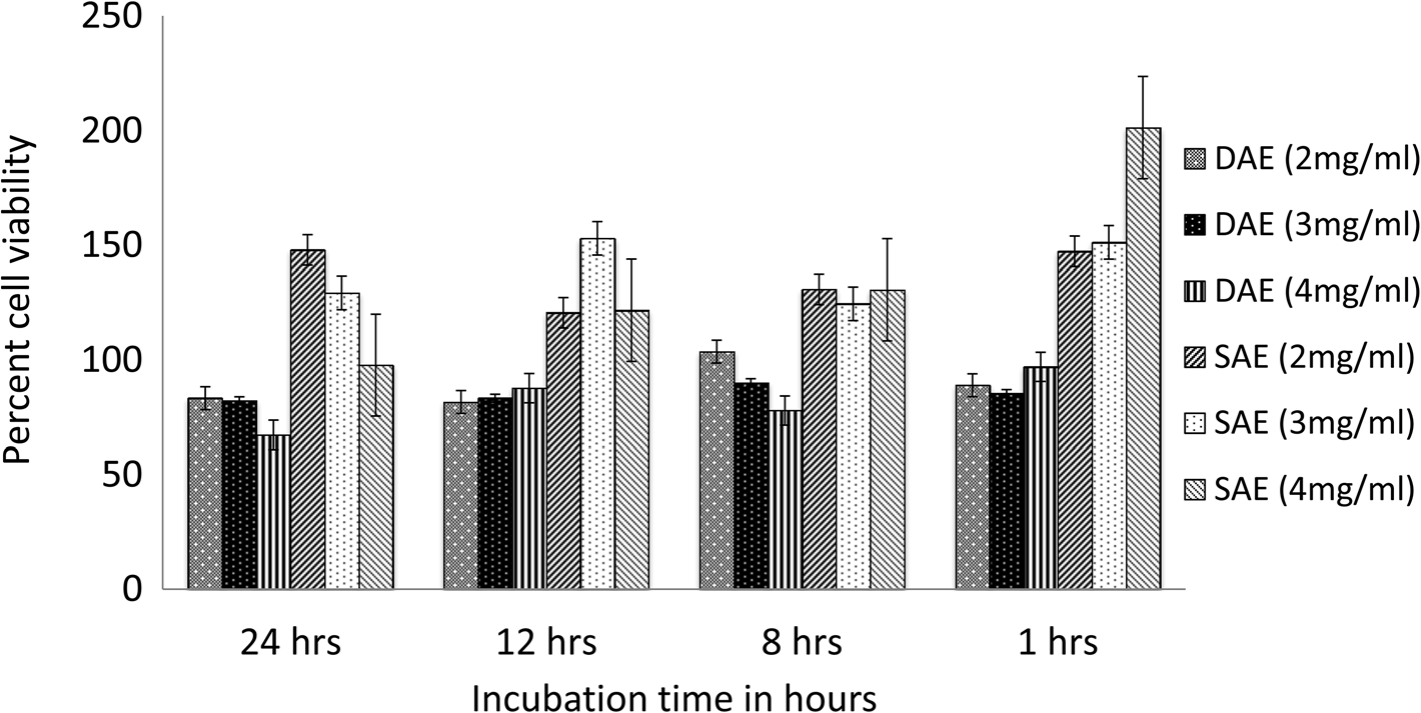
**Effect of DAE and SAE on Vero cell viability.** The effect of DAE and SAE on cell viability were significantly different at p < 0.05 level.

The extracts did not exhibit any significant cytotoxicity against Vero cell line at concentration ranging from 2 to 4 mg/ml after 24 hrs of incubation. The SAE exhibited less effect as compared to DAE (Figure 2). Three independent experiments were carried out and statistical significance was evaluated by Turkey test which is a single-step multiple comparison statistical test.

### Chemo-profiling of aqueous extract

HPTLC revealed presence of alkaloids, flavonoids, coumarins, saponins and steroids in the extract, however amides and phenolics were absent. The number of spots and the Rf values for each compound are represented in Table 3.

**Table 3:** **Chemo profile of sequential aqueous extract of *T. alternifolia *stem bark performed by HPTLC method**

**Compound**	**Number of spots observed**	**Rf values**
Alkaloids	2	0.77, 0.87
Flavanoids	11	0.03. 0.07, 0.35, 0.43, 0.5, 0.55, 0.6, 0.64, 0.68, 0.75, 0.8
Saponins	18	0.11, 0.17, 0.23, 0.25, 0.27, 0.34, 0.41, 0.45, 0.51, 0.53, 0.58, 0.63, 0.68, 0.84, 0.74, 0.56, 0.78, 0.84
Steroids	7	0.02, 0.04, 0.06, 0.15, 0.58, 0.78, 0.84
Coumarins	5	0.04, 0.07, 0.16, 0.57, 0.64

## Discussion

Plant derived medicines have been a part of traditional health-care in most parts of the world and the antimicrobial property of plant-derived compounds is well-documented [38]. For the first time here, we established the antimicrobial activity of crude aqueous extract of *T. alternifolia* against MRSA and VRSA. A study conducted by Duraipandiyan *et al.* in 2006, showed that the methanol and hexane extract of stem bark of *T. alternifolia* did not exhibit any antibacterial activity [25]. These observations related to activity may be attributed to the fact that different compounds from the plant material get extracted in solvents of different polarities. Moreover, the fact that the antimicrobial activity of both the SAE and DAE was similar (as indicated by the MIC values) demonstrates that the active principle in the plant stem bark was not extracted in less polar solvents like hexane, ether, methanol, ethyl acetate but got extracted in distilled water. During the extraction process the active principle within the extract survived boiling at 100°C for 6 hours suggesting that it is heat stable. Such a heat stable active principle has been previously reported from other plants [39,40].

Several studies have focused on establishment of antibacterial activity of the plant extracts but have not focused on showing the time dependent stability of its activity [22,23,39]. Here we report that the activity of the extract is stable over a period of 2 months after extraction. This indicates that the active principle present in the aqueous extract of *T. alternifolia* stem bark has a long shelf life in crude preparation. For a crude extract to be used for topical applications it is mandatory that the extract does not exhibit any cytotoxic activity. Although anticancer alkaloid camptothecin has been isolated from *T. alternifolia*; aqueous extracts in this study did not show presence of camptothecin (data not shown) [16]. The aqueous extracts did not exhibit any significant cytotoxic effect against Vero cell line (Figure 2); this is consistent with the absence of camptothecin in the extracts. On exposure to SAE, the viable cell counts of Vero cells were observed to be higher as compared to control, suggesting that SAE may have a probable positive effect on cell proliferation. The toxic compound/s present in the stem bark were probably extracted in less polar solvents during sequential extraction, thus, explaining the observed positive effect on cell proliferation only on SAE treatment.

The antimicrobial activity of the crude extract may be attributed to a specific compound or a combination of compounds. The knowledge about the chemical profile of the extract helps in explaining the observed activity and designing experiments for activity fractionation for isolation of the active principle. Alkaloids, flavonoids, coumarins, saponins and steroids are the compounds of plant origin known to have antibacterial activity. These compounds were detected in the SAE [40-44]. Hence further objective is to identify a potential lead compound, which can be developed into a candidate anti-infective drug, in particular for treatment of infections by multidrug resistant pathogens like MRSA and VRSA.

In the past few decades, MRSA have caused a major problem with nosocomial infections throughout the world [7]. In the developed countries, fluoroquinolones (ciprofloxacin and ofloxacin) are recommended for serious infections associated with *Staphylococci*[45] but vancomycin still remains the drug of choice for most MRSA-associated diseases [46]. In this study four MRSA isolates were resistant to vancomycin, five MRSA isolates were resistant and two were intermediately resistant to ciprofloxacin respectively, further emphasizing the difficulties in treatment of MRSA infections with antibiotics (Table 1). Moreover, in the last 2 decades, the number of new antimicrobial drugs that reached the marketplace has fallen to less than half the previous level [47]. Hence, more efforts should be directed towards the screening for new antimicrobial agents. The anti-MRSA activity exhibited by *T.alternifolia* stem bark extracts seems a promising step towards research for finding a new therapeutic agent against MRSA. However, extensive research needs to be carried out on this aspect of *T. alternifolia.*

## Conclusion

The aqueous extract of *T. alternifolia* has antibacterial activity against MRSA and VRSA but does not retain any cytotoxicity, which further validates the use of the plant in traditional medicine. The present study lays the basis for future research, to validate the possible use of *T. alternifolia* as a candidate in the treatment of MRSA infections.

## Competing interests

The authors declare that they have no competing interests.

## Authors’ contributions

MR and NM contributed equally towards the completion of manuscript. NM, MR, HK performed the experiments and analyzed the data. NM, MR, AP, SD and YS designed the study. NM and MR wrote the manuscript and SD, HK, AP and YS edited it. All authors read and approved the final manuscript.
